# Methodological procedure based on quantitizing/liquefying: a case study to assess work climate in an emergency department

**DOI:** 10.3389/fpsyg.2023.1247577

**Published:** 2023-12-22

**Authors:** Salvador Chacón-Moscoso, M. Teresa Anguera, Susana Sanduvete-Chaves, José A. Lozano-Lozano

**Affiliations:** ^1^Departamento de Psicología Experimental, Facultad de Psicología, Universidad de Sevilla, Seville, Spain; ^2^Departamento de Psicología, Universidad Autónoma de Chile, Santiago, Chile; ^3^Faculty of Psychology, University of Barcelona, Barcelona, Spain; ^4^Instituto de Ciencias Biomédicas, Universidad Autónoma de Chile, Santiago, Chile

**Keywords:** mixed methods, indirect observational methodology, in-depth interview, polar coordinate analysis, single-case study, work climate, emergency department, hospital

## Abstract

**Introduction:**

In the assessment of health organizations, results-based indicators are mainly used, with no consideration of internal work dynamics. This type of assessment forfeits much of the rich, useful information needed to make decisions on improving the organization. In order to address this, a rigorous procedure based on mixed methods is laid out here on gathering, analyzing, and interpreting data associated with the implementation process.

**Methods:**

A 55-year-old doctor was selected at random from among the staff who volunteered to be interviewed at the emergency department at a public hospital located in southern Spain for an interview. Qualitative data obtained from the in-depth interview (indirect observation) were progressively systematized (liquefied and quantitized) based on a theoretical framework until a code matrix was obtained, without losing or distorting any information. Afterwards, data quality was controlled using Cohen’s kappa (*κ*) coefficient. A quantitative polar coordinate analysis was then carried out using the free software HOISAN (v. 1.6.3.3) to obtain robust results, vectorizing the relationships between codes and specifying whenever such relationships were statistically significant (and if they resulted in behavior activation or inhibition). Finally, a supplementary quantitative and qualitative assessment was carried out.

**Results and discussion:**

The proposed method was applied to the needs assessment of teams in order to evaluate that work climate in the hospital’s emergency department Health Services of a hospital. Data quality control yielded an adequate result (*κ* = 0.82). Significant activation and inhibition of behaviors occurred, both prospectively and retrospectively. For instance, *We seek to understand the needs of our clients* and *We readily adapt to new circumstances* showed a significant activation (vector length = 3.43, *p* < 0.01) both prospectively (*Z*_sum_ = 0.48) and retrospectively (*Z*_sum_ = 3.4).

An adequate method to obtain detailed information about group dynamics in a work environment is presented, based on an in-depth interview. Practical applications for implementations to improve the functioning of organizations are presented.

## 1 Introduction

There have been three predominant theoretical approaches to the concept of work climate: (a) those that focus on the objective and structural characteristics of organizations (Li et al., 2022); (b) those that delve into the individual psychological characteristics of workers (Mozgovoy, 2022); and (c) those that adopt a perspective that considers both organizational and individual aspects (Altuntaş et al., 2021). This paper is based on the third approach, which is widely regarded as the most comprehensive given that it allows a multidimensional exploration of work climate.

In the context of healthcare services, the model proposed (Perry et al., 2005) conceptualizes work climate as an integral component of the organization primarily shaped by individual behavior and human interactions. According to this perspective, each working team within a hospital setting possesses a unique work climate, defined as the quality of the internal environment experienced by its members. It is believed to have a substantial influence on staff behavior. Therefore, any alteration in the work climate of a healthcare organization can lead to changes in other aspects of the organization, such as the effectiveness or quality of patient care (Santana and Pérez-Rico, 2023).

Hospital Emergency Departments (ED) exhibit distinctive features compared to other healthcare services. Professionals working in the ED confront any number of unique challenges: (a) they constitute the frontline of medical care for patients, playing a pivotal role in initial care (Trebach et al., 2021); (b) they provide care to a substantial volume of patients, irrespective of the severity of their condition (Holzinger et al., 2021); c) they deal with traumatic situations that require immediate and critical decision-making on a daily basis (Bijani et al., 2021); (d) they experience heightened levels of stress and responsibility owing to the inherent nature of their work (Barth et al., 2022); (e) they face a higher risk of post-traumatic stress disorder and depression due to their workplace experiences (Taylor et al., 2023). In this specific context, an assessment of work climate offers insights into both workgroup characteristics and organizational aspects, providing insight into job satisfaction, productivity, goal achievement, interpersonal relationships, and job performance (Sanduvete-Chaves et al., 2018; Lozano-Lozano et al., 2021).

Results-based indicators are mainly used when evaluating healthcare organizations, with no consideration of internal work dynamics. This type of assessment forfeits much of the rich, useful information needed to make decisions on improving the organization. Therefore, this work presents a rigorous procedure based on mixed methods to gather, analyze, and interpret the data associated with the implementation process. Qualitative data obtained from an in-depth interview (indirect observation) were progressively systematized until a code matrix was obtained, without losing or distorting any information. A quantitative polar coordinate analysis was then carried out to obtain robust results, vectorizing the relationships between codes. Finally, a supplementary quantitative and qualitative assessment was carried out.

Observational methodology was optimal for this task, given that it is both rigorous and flexible (Chacón-Moscoso et al., 2014; Anguera, 2020). Specifically, indirect observation was used (Anguera, 2021). In terms of the information obtained in the in-depth interview, as noted in an earlier work,

*…all these materials constitute an extremely rich source of information for studying everyday life, and they are continuously growing with the burgeoning of new technologies for data recording, dissemination, and storage. Narratives are an excellent vehicle for studying everyday life, and quantization is proposed as a means of integrating qualitative and quantitative elements. However, this analysis requires a structured system that enables researchers to analyze* var*ying forms and sources of information objectively* (Anguera et al., 2018c).

Indirect observation facilitates the analysis of diverse texts, as has been shown in works on self-registrations (Squires et al., 2011), teacher – student conversations (García-Fariña et al., 2018), therapist – teenager patient dialog (Arias-Pujol and Anguera, 2020), therapist – adult patient dialog (del Giacco et al., 2020), in-depth interviews with elite trainers (Nunes et al., 2022), in-depth interviews with professional coaches and players (Iván-Baragaño et al., 2023), panel discussions with professionals about the foreseeable consequences of COVID-19 (Moya et al., 2020), and in-depth interview with family firms where there is an intrafamilial conflict (Alvarado-Alvarez et al., 2021).

In-depth interviews, unlike other types of interviews, allow researchers to delve in detail into aspects that required the participant to recount them as they were experienced. Therefore, these interviews capture a wide range of opinions and experiences among a group of professionals. They provide more extensive and detailed data on the unique contextual factors of each participant, facilitating a comprehensive representation of professionals’ daily experiences, their decision-making processes, and their interactions with both patients and the medical team (Merriam and Tisdell, 2016; Marshall et al., 2022). Furthermore, in-depth interviews can be adapted, including new questions or modifying existing ones based on the previous responses of participants. This enables a deeper exploration of specific areas of interest (Rubin and Rubin, 2005).

A single-case study can be defined as an intensive study of a single unit with an aim to generalize across a larger set of units (Gerring, 2004; Hunziker and Blankenagel, 2021). The interest in single-case studies has increased in recent years in different health fields (Stetler, 2009; Champagne, 2014; Porcheret, 2014). Single-case studies present advantages, such us they enable methodological advances, especially in the case of observational studies (Kitzmiller, 2010; Arias-Pujol et al., 2022). Additionally, the polar coordinate analysis proposed has been used in recent years in various fields, such as clinical psychology (Rodríguez-Medina et al., 2018; del Giacco et al., 2020; Arias-Pujol et al., 2022), occupational health (Portell et al., 2019), classroom activities (Belza et al., 2019), sports (Maneiro et al., 2020 Iván-Baragaño et al., 2023), and conflict mediation (Alvarado-Alvarez et al., 2021). However, the context in which this single-case study (Sandelowski et al., 2009; Anguera, 2018; Arias-Pujol et al., 2022) was developed, a hospital ED, is totally new.

Though the approach to single-case studies has generally been qualitative, it has also been heterogeneous in the literature. Our objective was to develop a case study from a mixed methods approach, thus integrating qualitative and quantitative elements. Specifically, a needs assessment (Sanduvete-Chaves et al., 2009) of work teams was carried out to evaluate the work climate. Given that the starting point was a single interview, an intensive treatment of the information is needed.

## 2 Methods

Two checklists guided the methodological process: the Guidelines for Reporting Evaluations based on Observational Methodology (Portell et al., 2015) and the Methodological Quality Checklist for studies based on Observational Methodology (Chacón-Moscoso et al., 2019), derived from the Guidelines for Reporting Evaluations based on Observational Methodology, which presents adequate validity evidence based on its content (Chacón-Moscoso et al., 2018).

### 2.1 Design

The observational design presented is idiographic, punctual, and multidimensional (Blanco-Villaseñor et al., 2003; Anguera et al., 2021). It is idiographic because it is based on a single case (an interview). The design can be considered punctual because the information was obtained in a single session, although there was intra-session follow-up because the full interview was analyzed. Finally, it is multidimensional because the study was based on a theoretical framework and structured in four dimensions. It then materialized in the observation tool.

### 2.2 Participant

The person selected at random from among the staff who volunteered to be interviewed was an ED worker at a public hospital in the south of Spain. The patients at this hospital hail from a diverse demographic. The selection criteria for the participant were (a) currently working at the ED when the study was carried out, and (b) having worked in the ED for at least three years.

The worker interviewed was a 55-year-old man who participated voluntarily. He was a doctor in the ED with an indefinite contract, 25 years of experience in his profession, and 23 years working at his current workplace. One of the authors, JALL, interviewed the participant in a hospital room. The interview yielded substantial new and rich information.

### 2.3 Instruments

#### 2.3.1 Observation instrument

Based on a theoretical framework that laid out the fundamental work climate factors for ED teams (Sanduvete-Chaves et al., 2018) and the unique characteristics of this service, a non-standard tool was designed in keeping with indirect observation (Portell et al., 2015; Anguera et al., 2018c; Anguera, 2021). This tool combined field format and a system of categories.

The dimensions proposed for the theoretical framework included (A) job satisfaction, referring to how workers feel about their job and work conditions; (B) productivity/achievement of aims, which refers to the workers’ sense of whether they have everything they need to do their job properly and achieve their goals; (C) interpersonal relationships, referring to the workers’ feelings about their fellow team members; and (D) work performance, referred to all aspects related to how the workers do their jobs (Table 1). A system of exhaustive and mutually exclusive categories was built for each dimension.

**Table 1:** Indirect observation instrument.

Dimensions	Category systems	Code
A. Work satisfaction	We take pride in our work	A1
	We seek to understand the needs of our clients	A2
	We readily adapt to new circumstances	A3
	We strive to achieve successful outcomes	A4
	We have the experience to do our job well	A5
	Our workday provides the time we need to do our jobs	A6
	We coordinate well with the other hospital services	A7
	Each member has a role based on his/her area of expertise	A8
	Our work is important	A9
	We develop our skills and knowledge	A10
B. Productivity/achievement of aims	Our work group is known for quality work	B1
	We have a common purpose	B2
	We have the necessary infrastructure	B3
	We receive the necessary training	B4
	The characteristics of our service are appropriate	B5
	Our services are provided correctly	B6
	Our work group is known for its productivity	B7
	We feel motivated doing our work	B8
	The merit of the job we do is recognized	B9
	Our colleagues value our profession	B10
	The work we do is appreciated	B11
	Our expertise is recognized	B12
	Our expectations have been fulfilled	B13
	The type of patient we see is aligned with the services we provide	B14
	We know our patients	B15
	We coordinate with other hospital services	B16
	We are recognized for our individual contributions	B17
	We have a plan that guides our activities	B18
	We participate in the decisions of our group	B19
	We know what is expected of us on the job	B20
C. Interpersonal relationships	We have good communication within the group	C1
	Team members have a good working relationship	C2
	I feel comfortable with my work group	C3
	I have good personal relationships	C4
	The work climate is conducive to group work	C5
D. Performance at work	We understand each other’s capabilities	D1
	I know my professional shortcomings	D2
	We know what tasks each member handles	D3
	The patients we see are aligned with the services we provide	D4

#### 2.3.2 Recording device

A digital audio recorder was used to record the in-depth interview. Express Scribe software (version 2.0) was used for the transcription.

### 2.4 Procedure

#### 2.4.1 Quantitizing/liquefying

According to the methodological process of indirect observation, the text can be liquefied (Anguera, 2020). This is combined with the quantitizing of the text in keeping with (direct and indirect) observational methodology, from the mixed-method perspective. It is possible to operate quantitatively with codes to which the textual units are assigned, either uniquely or in co-occurrence.

The mixed methods (Creswell and Plano Clark, 2011), broadly developed in the last two decades, offers vast potential for the integration of qualitative and quantitative elements. When referring to such integration paths, connecting (Creswell and Plano Clark, 2011) was chosen to build one dataset upon the other. The connecting forms the groundwork for the quantitizing, which consolidates previous proposals that have enabled the observational methodology to be considered a mixed method on its own (Anguera and Hernández-Mendo, 2016 Anguera et al., 2017). Neither methods nor techniques are combined, as is traditional according to conventional mixed methods or in multimethod studies (Anguera et al., 2018b). Data from different sources are neither combined. The information is transformed in a different way than what is described in the literature on mixed methods (Sandelowski et al., 2009).

**Figure 1:**
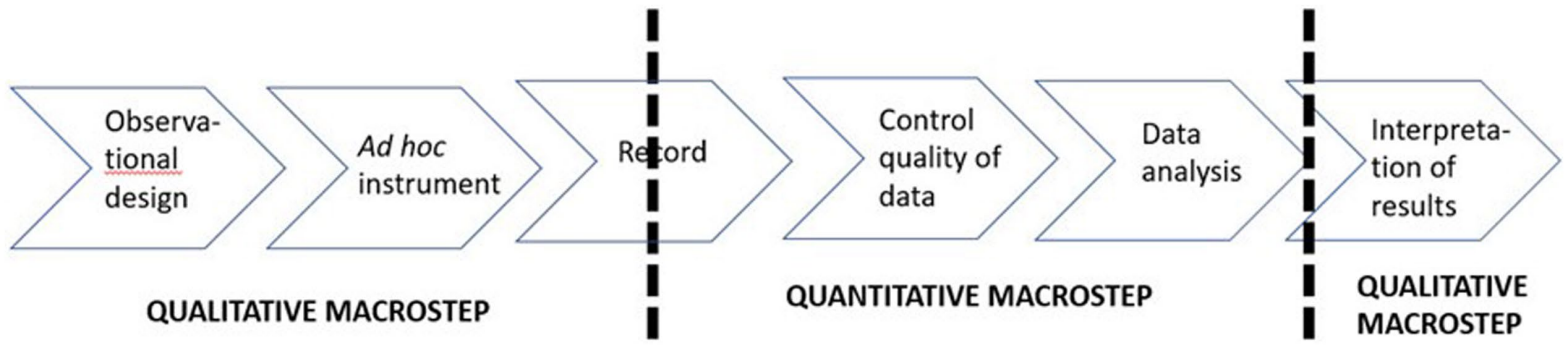
Steps of indirect observation.

This work presents a proposal for quantitizing that can be adapted to all situations, enabling the observational methodology to be used as a scientific method (Anguera et al., 2018a Chacón-Moscoso et al., 2021) Additionally, it fits with the connecting (Creswell and Plano Clark, 2011). It materializes when the macro-stages QUAL-QUAN-QUAL are alternated (Figure 1). In the first QUAL stage, the observational design is proposed, and the indirect observation instrument is built, yielding a descriptive record that is systematized until it becomes a code matrix. In the QUAN stage, the systematized record is subjected to data quality control (Chacón-Moscoso et al., 2023), using robust analytical techniques appropriate for categorical data. Finally, in the second QUAL stage, the results are interpreted. This procedure is consistent with the nature of the information obtained and the methodology applied.

The information captured during the interview is transformed into a record and this constitutes the raw material for analysis. This data collection relies on a text transcription as its first step, taking into account that “transcripts are necessarily selective in the details that are represented” (Hepburn and Bolden, 2013). In this study, only verbal behavior was considered, without taking into account any additional information that could be garnered (gesture, gaze, cadence, etc.) in studies focused on the multimodality of human behavior.

The first decision to establish was the segmentation criterion in units. The behavior flow was set in interactive units (Birdwhistell, 1970) that were then ranked based on their granularity (Schegloff, 2000). Various levels of granularity could be formed (Schegloff and Sacks, 1973). Given the advantages and disadvantages of each unit type, we have combined syntactic and contextual criteria (Anguera, 2020).

**Figure 2:**
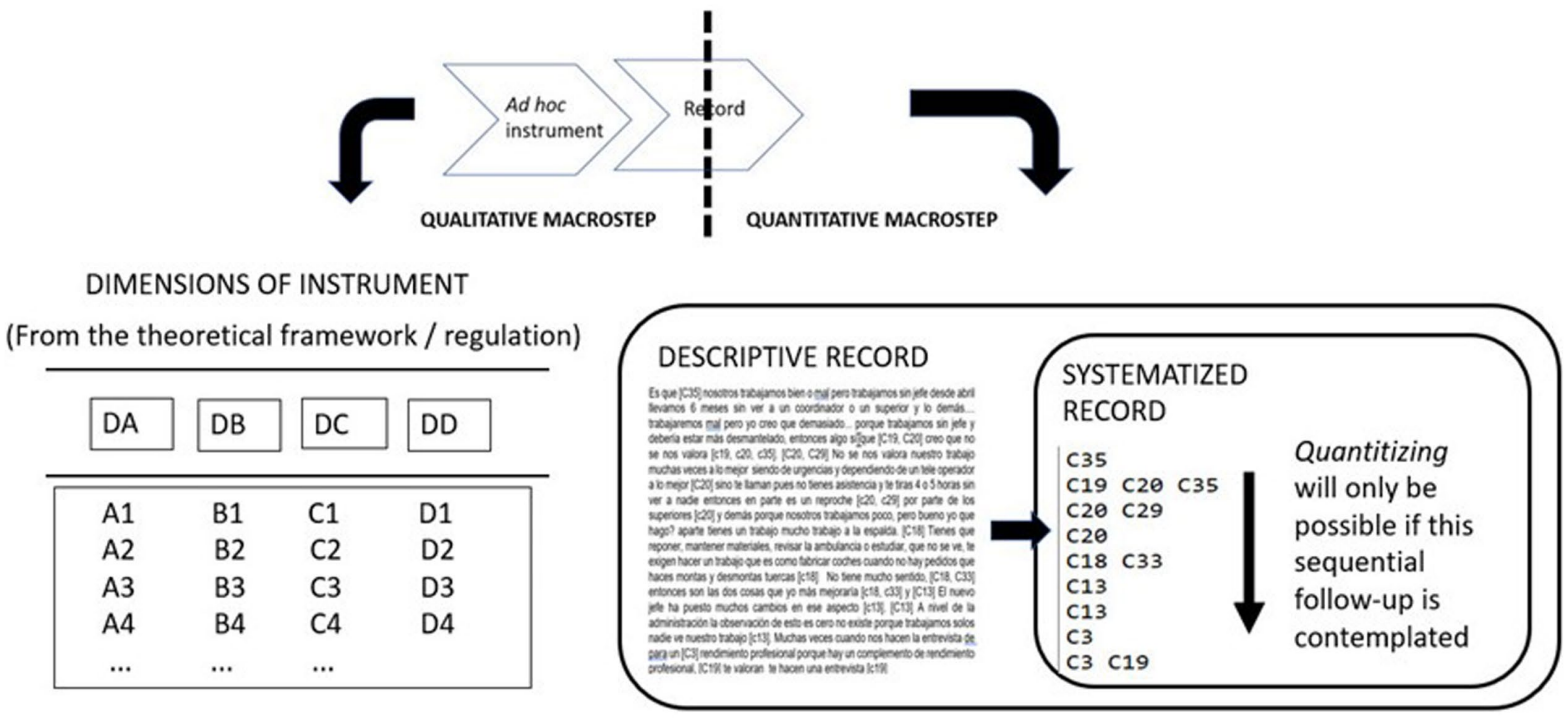
A graphic depiction of quantitizing/liquefying.

Once the transcription was segmented into text units by means of the indirect observation instrument (Table 1), each text unit was coded. One or more codes were obtained for each of them, depending on the corresponding dimension/s involved. If only one dimension was affected, the text unit was assigned to the corresponding category; if several dimensions were affected, the co-occurring categories were recorded, generating a systematized record (Figure 2). The key to this coding is based on the data classification widely used in both direct and indirect observational methodology (Bakeman, 1978; Anguera et al., 2018c), from the beginning to the end of the session, obtaining a codes matrix whose columns were the four dimensions (A, B, C and D) established in the indirect observation instrument. Each row represented one of the textual units obtained when the text of the interview was segmented.

When completed, the first QUAL stage yielded a code matrix. Since data are already arranged sequentially and available for analysis, once the data went through quality control, quantizing is possible.

#### 2.4.2 Data quality control

To control the data quality, (Holgado-Tello et al., 2016) three independent researchers (authors MTA, SCM, and SSC) coded the transcription of the interview, dividing the text into content units and assigning a code for each different content. Disagreements were resolved through consensus. The Supplementary material includes a segment of the interview with an example of a coding assignation. The kappa (*κ*) coefficient (Cohen, 1960) was calculated using HOISAN (v. 1.6.3.3), based on the records made by the three coders. Values over 0.7 were considered adequate.

#### 2.4.3 Polar coordinate analysis

Polar coordinate analysis is one of the most suitable categorical data analysis techniques (Blanco-Villaseñor et al., 2003; Anguera et al., 2021), given that it allows a relations map to be drafted. This map shows the statistical relationships of association between a specific behavior of interest, the focal behavior, and others known as conditioned behaviors, i.e., behaviors that may be related to the focal behavior. For those that do relate, it is then necessary to determine the type and degree of the relationship. This quantitative relations analysis also allows these relationships to be charted as vectors, facilitating conclusions about the relationships, types, and intensities of behaviors.

The polar coordinate analysis (Sackett, 1980) constitutes a second stage of the lag sequential analysis (Bakeman, 1978). It considers the adjusted residuals obtained in the lag sequential analysis as data, once these are corrected (Allison and Liker, 1982). The analysis starts with the focal behavior and is both prospective (forward-looking) and retrospective (looking backwards). It provides insight into how the relationship between the focal behavior and the conditioned behaviors varies or evolves over time (in this case, throughout the recording of the interview). Retrospectivity from lag 0 to −1, from lag −1 to −2, and so on, was applied (Anguera, 1997). This analysis algorithm was included when programming the HOISAN software (Hernández-Mendo et al., 2012).

The data obtained were independent because each datum is calculated based on the corresponding lag. This enabled the use of parameter *Z*_sum_ or the “flag” parameter (Sackett, 1980), thus facilitating data reduction. The *Z*_sum_ parameter is based on the principle that the distribution of the sums of n-independent *Z* values is normal, with *μ* = 0 and *σ* = 1. It is calculated using the formula


$$Z_{\mathrm{sum}}=\frac{\sum Z}{\sqrt{n}}$$

where *n* is the number of lags. *Z*_sum_ is calculated for each conditioned behavior. The prospective *Z*_sum_ values are obtained considering the positive lags, and the retrospective *Z*_sum_ values are calculated considering the negative lags. The number of positive and negative lags should be the same, and a minimum of five lags is recommended, i.e., from −1 to −5 and from +1 to +5 (Sackett, 1980).

For the prospective and retrospective *Z*_sum_ values, the relationships between focal behavior and conditioned behaviors are vectorized (Sackett, 1980). The length or radius of each vector would be


$$\mathrm{Length}=\sqrt{{\left(Z_{\mathrm{sum}\;\mathrm{prospective}}\right)}^{2}+{\left(Z_{\mathrm{sum}\;\mathrm{retrospective}}\right)}^{2}}$$

and the angle


$$\varphi =\mathrm{Arc}\;sen\frac{Z_{\mathrm{sum}\;\mathrm{retrospective}}}{\mathrm{Length}}$$

A vector is obtained for each conditioned behavior. The graphic origin of all vectors will be the focal behavior. Given that prospective and retrospective *Z*_sum_ values can be positive or negative, the corresponding vectors can be plotted considering that the prospective *Z*_sum_ are represented on the abscissa axis, and the retrospective *Z*_sum_, on the order axis.

The adjusted residuals, *Z* values, angles, and vector lengths are obtained using HOISAN (v. 1.6.3.3) [*www.menpas.com*] (Hernández-Mendo et al., 2012), which calculates them from sequential multi-event type II data (Bakeman, 1978). In addition, the vector graphing was optimized using the free software R (Rodríguez-Medina et al., 2022) [https://jairodmed.shinyapps.io/HOISAN_to_R_2022/].

The vectors are interpreted based on whether the prospective and retrospective *Z*_sum_ values obtained are positive or negative, as indicated in Table 2. The significant relationships between the focal behavior and the conditioned behaviors (*p* < 0.05) are characterized by vector lengths >1.96, while highly significant relationships (*p* < 0.01) are distinguished by vector lengths >2.58.

**Table 2:** Interpretive guideline for vectors in polar coordinate analysis.

Quadrant in which the vector of each conditioned behavior is located	Sign of the prospective *Z*_sum_	Sign of the retrospective *Z*_sum_	Meaning
I	Positive	Positive	Prospective and retrospective activation
II	Negative	Positive	Prospective inhibition, retrospective activation
III	Negative	Negative	Prospective and retrospective inhibition
IV	Positive	Negative	Prospective activation, retrospective inhibition

## 3 Results

Intercoder reliability was *κ* = 0.82, which indicated satisfactory concordance. Afterwards, a polar coordinate analysis was carried out considering each code of the indirect observation instrument as a focal behavior and all the codes that comprise the instrument as conditioned behaviors.

**Figure 3:**
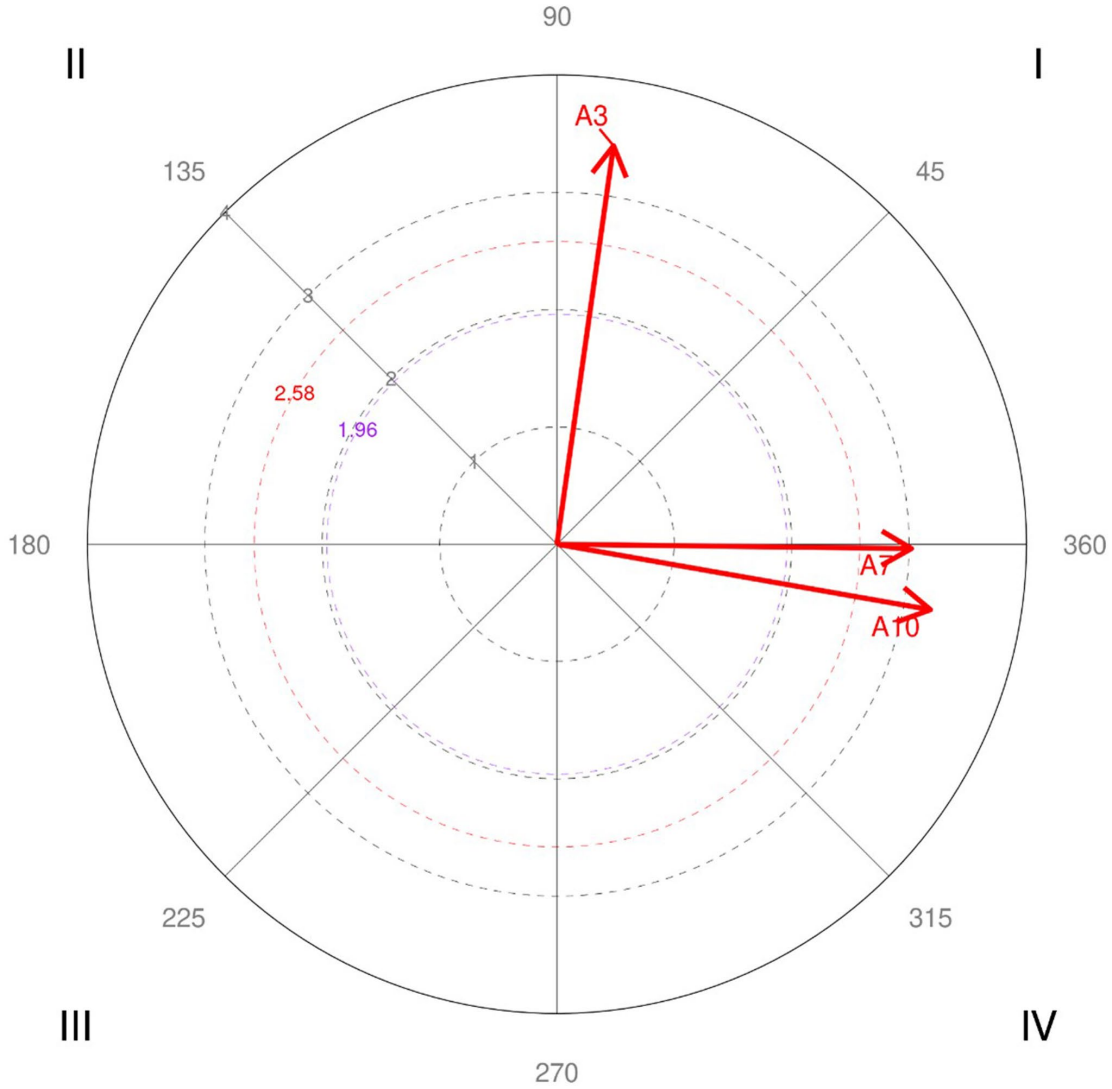
Significant vectors obtained in the polar coordinate analysis, considering A2 as a focal behavior, and all codes as conditioned behaviors.

**Figure 4:**
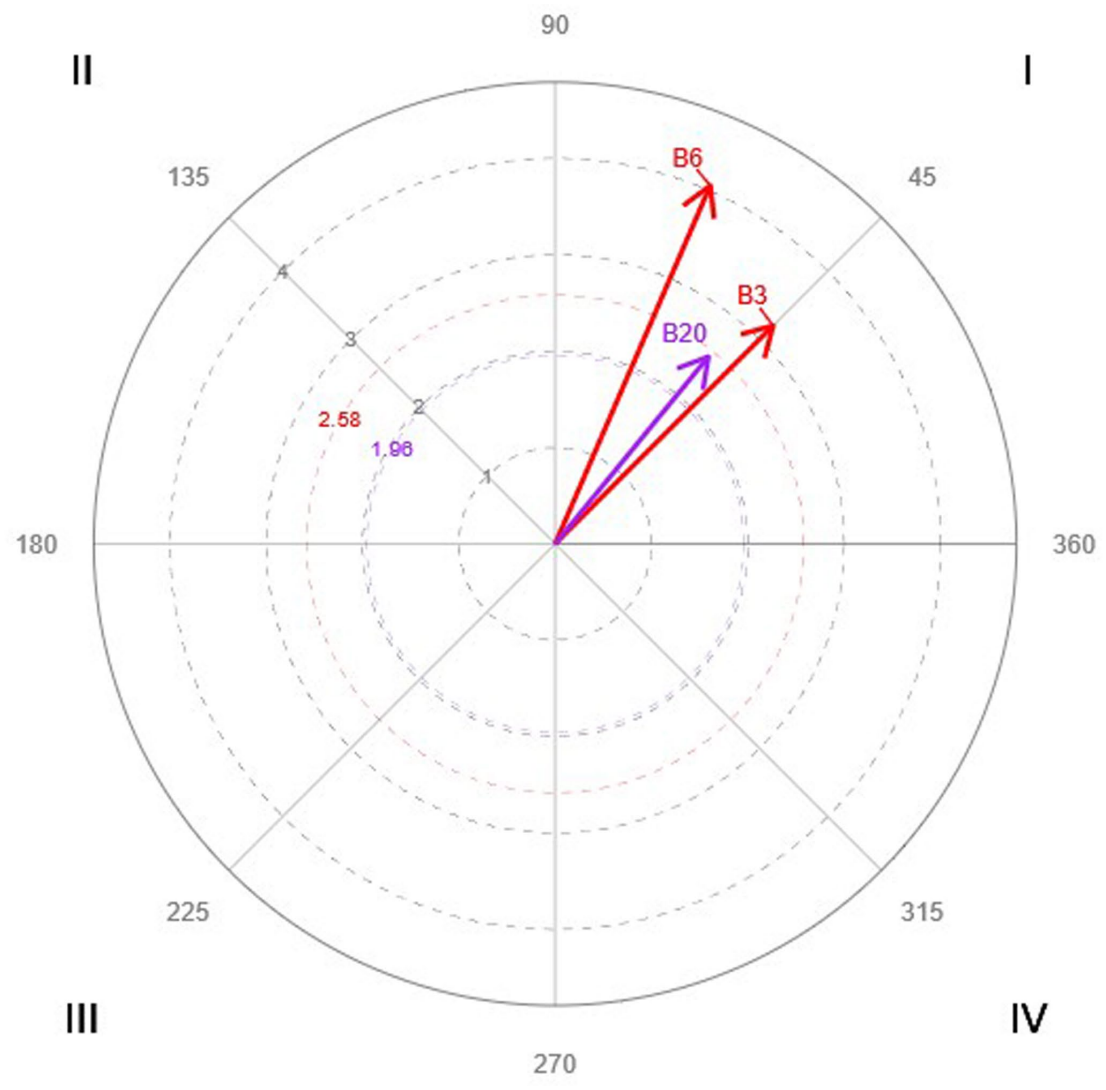
Significant vectors obtained in the polar coordinate analysis, considering B2 as a focal behavior, and all the codes as conditioned behaviors.

The results corresponding to the focal behaviors A2 *We seek to understand the needs of our clients* (Table 3 and Figure 3), and B2 *We have a common purpose* (Table 4 and Figure 4) are presented for illustrative purposes and considering all the codes of the indirect observation instrument as conditioned behaviors. Tables 3, 4 present all results, regardless of whether the relationship was significant. Figures 3, 4 include only significant and highly significant vectors.

**Table 3:** Parameters corresponding to the polar coordinate analysis, considering A2 as a focal behavior.

Category	Quadrant^a^	Prospective *Z*_sum_	Retrospective *Z*_sum_	Ratio	Vector length	Vector angle
A2	I	0.31	0.31	0.71	0.44	45
A3	I	0.48	3.4	0.99	3.43**	81.92
A4	II	−0.42	1.29	0.95	1.36	108.11
A5	I	0.31	0.45	0.83	0.55	55.78
A6	III	−1.34	−0.53	−0.36	1.44	201.34
A7	IV	3.02	−0.04	−0.01	3.02**	359.3
A9	II	−0.56	1.31	0.92	1.43	112.97
A10	IV	3.18	−0.56	−0.17	3.23**	350.05

All codes were considered conditioned behaviors.

a Quadrant where the vector is located.

**p < 0.01.

**Table 4:** Parameters corresponding to the polar coordinate analysis, considering B2 as a focal behavior.

Category	Quadrant^a^	Prospective *Z*_sum_	Retrospective *Z*_sum_	Ratio	Length	Angle
B1	III	−0.3	−0.3	−0.71	0.43	225
B2	III	−0.17	−0.17	−0.71	0.25	225
B3	I	2.26	2.26	0.71	3.2**	45
B4	III	−0.65	−0.65	−0.71	0.92	225
B5	III	−0.25	−0.25	−0.71	0.35	225
B6	I	1.61	3.72	0.92	4.05**	66.57
B7	III	−0.43	−0.43	−0.71	0.61	225
B8	III	−0.25	−0.25	−0.71	0.35	225
B9	III	−0.35	−0.35	−0.71	0.5	225
B10	III	−0.17	−0.17	−0.71	0.25	225
B11	III	−0.17	−0.17	−0.71	0.25	225
B13	III	−0.17	−0.17	−0.71	0.25	225
B14	III	−0.25	−0.25	−0.71	0.35	225
B15	III	−0.43	−0.43	−0.71	0.61	225
B16	III	−0.35	−0.35	−0.71	0.5	225
B18	III	−0.47	−0.47	−0.71	0.66	225
B19	III	−0.39	−0.39	−0.71	0.56	225
B20	I	1.59	1.94	0.77	2.51*	50.66

All codes were considered conditioned behaviors.

a Quadrant where the vector is located.

*p < 0.05; **p < 0.01.

When A2, *We seek to understand the needs of our clients*, is considered the focal behavior, it is found to have a significant relationship with A3, *We readily adapt to new circumstances*. There is reciprocal activation A2 ↔ A3 (quadrant I), showing its functional and organizational benefits for the ED. Additionally, asymmetric relationships with A7, *We have good relations with the other services*, and A10, *We develop our skills and knowledge*, were found in quadrant IV. A2 favors both behaviors (prospective *Z*_sum_ = 3.02 and 3.18 respectively). Nevertheless, A7 and A10 present an inhibitory relationship with A2 close to zero (retrospective *Z*_sum_ = −0.04 and − 0.56 respectively); i.e., they have a good relationship with the other hospital services or developing skills and knowledge does not imply that it is easy to adapt to new circumstances.

Considering B2, *We have a common purpose*, as a focal behavior, three significant reciprocal activations (located in quadrant I) are found with the following behaviors: B3, *We have the necessary infrastructure* (B2 ↔ B3); B6, *Our service works correctly* (B2 ↔ B6); and B20, *We know what is expected of us on the job* (B2 ↔ B20). This result is especially relevant from the point of view of shared mission and good organization.

## 4 Discussion

In-depth interviews are not commonly used in the implementation of programs, given that research has not indicated any productive use of them. This paper presents an adequate method to obtain detailed and thorough information based on an in-depth interview.

The mixed method approach used herein started exclusively with qualitative data (the responses to an in-depth interview) which, after being systematized, were subject to a quantitative analysis, and finally reached a qualitative interpretation of the results. Polar coordinate analysis is commonly used in direct observation. This is the first time to our knowledge that its use is presented in indirect observation and in the context of ED.

There are some limitations to the method proposed. First, substantially more time and human resources are needed for an in-depth interview than for other tools, such as questionnaires (Cano-García et al., 2017). Nevertheless, we consider that the level of detail and usefulness of the information obtained proved worthwhile. Additionally, the qualitative interpretation could prove difficult for an external evaluator, given that this person is unfamiliar with the context and details. For this reason, we recommend carrying out an internal evaluation within the organization or, if an external evaluation is needed, to work closely with stakeholders from the organization that is being evaluated.

All workers in the ED were asked to participate. As a result, we interviewed different ED professionals (emergency technicians, nurses, doctors, administrative staff and security personnel). Nonetheless, the aim of the present study was to show how qualitative and quantitative elements can be integrated through indirect observation, enabling a mixed-method approach to be used in an in-depth interview. In order to achieve this, a doctor’s interview was chosen at random from the set of interviews conducted and analyzed as an illustrative but non-representative example (Gerring, 2006). High external validity was not the main objective in this study, which is why we do not discuss the generalization of the results. It is impossible to know whether a single doctor is not representative of the doctor subgroups unless the homogeneity of the subgroup was assessed beforehand. Additionally, other ED workers would probably have a different point of view about their group’s work climate than doctors do. To obtain representativeness of the work climate in this ED, a study with multiple cases would be useful: other workers, such as nurses and orderlies, would be included, and the kappa coefficient would indicate the extent of agreement between their responses.

There are some important practical applications for implementation systems in terms of improving the functioning of organizations. Taking the case study presented herein, and considering that B2, *We have a common purpose*, and B6, *Our service works correctly*, presented significant reciprocal activation, workers should receive a clarification about the common purpose of the services their area provides if it is not deemed to be working properly (for example, if patients complain about the treatment received).

In the single-case study presented, the method proposed was illustrated in the context of an ED. Nevertheless, the method is flexible and can be adapted to contexts such as another hospital service and any other organization, as long as an informative in-depth interview can be carried out.

## Data availability statement

The raw data supporting the conclusions of this article will be made available by the authors, without undue reservation.

## Ethics statement

The studies involving humans were approved by Scientific Ethics Committee of Universidad Autónoma de Chile. The studies were conducted in accordance with the local legislation and institutional requirements. The participants provided their written informed consent to participate in this study.

## Author contributions

SC-M and MTA came up with the idea for the study. SC-M, MTA, and SS-C contributed to the design. JL-L acquired the data. MTA and JL-L conducted the analysis. All authors interpreted the results, wrote the initial draft of the manuscript, revised the article and approved the final version. All authors contributed to the article and approved the submitted version.
