# Supplementary material for: Methodological procedure based on quantitizing/liquefying: a case study to assess work climate in an emergency department
Source: Front Psychol. 2023 Dec 22;14:1247577. doi: 10.3389/fpsyg.2023.1247577 (PMC10774222; doi:10.3389/fpsyg.2023.1247577)
Supplement: Supplementary file 1 [file Table_1.DOCX]

Supplementary Material

# Supplementary Data. Coding of an extract of the interview.

The interviewee is given a short introduction about what the interview will consist of, the reason for the interview and its purpose, with the aim of being as least directive as possible in its development.

[B7] Comparing is impossible. It is impossible, because there are so many exclusion factors and, whenever they compare me, they compare us, because they always compare us [B7], it could be something weighted, establishing some weighted values, it could be weighting some criteria and establishing some weightings. Then, you will be able to more or less compare, but not compare one with the other (No, the first approach we are going to do is qualitative and the second quantitative).

(The first thing I would like you to tell me is the time you have been working in the service and the functions you do). [A5] Okay, well, I have been working in the service for four years and... a little more than four years [A5], in the mobile team, eh?, [A3] as such, how I started working, how they proposed the position to me, how a vacancy became available as the mobile team [A3] and [C2] I had several colleagues within already working and so on and... well, they convinced me, they convinced me and so on [C2], [A3] is a very different job. I am a family doctor and mine is primary care and so on, and the world is very different and so on. It totally has nothing to do with hospital care nor... does it have very specific peculiarities [A3]. [A3] Nothing more than work because you work on the street with many external conditions, apart from health issues. Assistance has more social and environmental problems. Manage the setting, or treat a traffic accident, or go to an aggressive home in a conflictive neighborhood [A3], or you are, ah... In [B15] the hospital, in the emergency room, the patient's family can be conflictive but, in the house, the territory is theirs and you have not one, but twenty relatives, and people are stubborn and you... you are much more..., more susceptible, right? [B15]. But [C2] well, my colleagues and others convinced me a little, and very well, very well. The truth is that the best thing I have is the team, the colleagues [C2], [C5] the excellent environment [C5]. [C3] We all knew each other before, except one. And... well, it was the main reason why I entered; because of their references and because they were there, and internally this is the best we have [C3]. [B3] All mobile teams work with very limited material, we go with... if there is any drug, we carry three vials and, if three are used, there is no other; so you don't look in the hospital... you don't look for anything, because that's what you have. So you have to take three and if you spend one and have to put another one, then immediately, because there is no other. It's like ammunition: either you do it this way or you have nothing to shoot with [B3]. [A4] So, you are on the street, you are sold, and you have to do it, and then that, you are in a world that is very... on top and, perhaps, very concentrated because if not, perhaps the next day you sink your partner [A4].

(And specific years in the service...) [A5] on January 1, 2000, I started; Wow, I started in the new year; It's exactly how long it took [A5].

(And the functions...) [D3] The functions are extra-hospital care, with the mobile team we have home care, everything that is extra [D3], [A7] outside the health centers; We cover both health centers and hospitals; public roads, all homes, institutions, police station, nursing home, port, prison, eh? ...schools, institutes, everything, private homes, and public roads, including urban roads, interurban roads, and everything because we go from beach rescue with immigrants to hikers who have fallen down a lane or a path; we cover it all [A7].
